# 

**Published:** 1875-12

**Authors:** 


*CONTENTS:*

| | PAGE |
|---|---|
| Taming the Appetite | 375 |
| Whole Wheat Flour | 376 |
| A Deadly Poison | 376 |
| Is Cancer Curable | 377 |
| Advertised Medicines | 378 |
| Our Summer at Maplewood. (Concluded.) Chapter XI, Little Things | 379 |
| Bad Feet | 388 |
| Chronic Diseases | 389 |
| Killing by Contraction | 390 |
| Man and other Animals | 291 |
| A Plan for Getting Work | 392 |
| Care for the Eyes | 393 |
| Sewer Gas | 394 |
| Soothing and Healing | 396 |
| Alchohol and Longevity | 397 |
| Reason and Insanity | 398 |
| City of Hygeia | 399 |
| Keep your Feet Warm | 400 |
| Health Food Company | 401 |
| A Sleepless Guardian | 403 |
| Analysis of Ale | 404 |
| Making a Home | 405 |
| A Lucky Man | 407 |
| A Vulnerable Point | 408 |
| Cast Iron Heating Devices | 409 |
| What shall we Drink | 410 |
| Good Way to Cook Apples | 411 |
| Chapped Hands | 411 |
| Early Influences | 412 |
| To Parents | 412 |
| Economy | 412 |
| A Wonderful Penman | 413 |
| Book Reviews | 413 |
| A Victim of Dissipation | 414 |
| Lifting for Health | 415 |
| Sometime | 416 |

